# Integrated analysis of circRNA-associated ceRNA network in ischemic stroke

**DOI:** 10.3389/fgene.2023.1153518

**Published:** 2023-05-30

**Authors:** Rongli Fan, Die Hu, Maiqiu Wang, Huilin Zheng, Yifeng Zhou, Lei Zhang

**Affiliations:** ^1^ Zhejiang Provincial Key Laboratory for Chemical and Biological Processing Technology of Farm Products, Department of Biological and Chemical Engineering, Zhejiang University of Science and Technology, Hangzhou, Zhejiang, China; ^2^ Department of Information and Electronic Engineering, Zhejiang University of Science and Technology, Hangzhou, Zhejiang, China

**Keywords:** whole transcriptome-wide analysis, ischemic stroke, ceRNA network, hsa_circ_0011474, CDKN1A

## Abstract

**Introduction:** Stroke, of which ischemic stroke (IS) is the major type, is the second leading cause of disability and death worldwide. Circular RNAs (circRNAs) are reported to play important role in the physiology and pathology of IS. CircRNAs often act as competing endogenous RNA (ceRNA) to regulate gene expression by acting as miRNA sponges. However, whole transcriptome-wide screenings of circRNA-mediated ceRNA networks associated with IS are still lacking. In the present study, we constructed a circRNA-miRNA-mRNA ceRNA network by whole transcriptome-wide analysis.

**Methods:** CircRNAs, miRNAs and mRNAs expression profiles were downloaded from the Gene Expression Omnibus (GEO) datasets. We identified differentially expressed (DE) circRNAs, miRNAs, and mRNAs in IS patients. StarBase and CircBank databases were used to predict the miRNA targets of DEcircRNAs, and mirDIP database was used to predict the mRNA targets of DEmiRNAs. CircRNA-miRNA pairs and miRNA-mRNA pairs were established. Then, we identified hub genes via protein-protein interaction analysis and constructed a core ceRNA sub-network.

**Results:** In total, 276 DEcircRNAs, 43 DEmiRNAs, and 1926 DEmRNAs were explored. The ceRNA network included 69 circRNAs, 24 miRNAs, and 92 mRNAs. The core ceRNA subnetwork included hsa_circ_0011474, hsa_circ_0023110, *CDKN1A, FHL2, RPS2, CDK19, KAT6A, CBX1, BRD4*, and *ZFHX3*.

**Discussion:** In conclusion, we established a novel hsa_circ_0011474 - hsa-miR-20a-5p/hsa-miR-17-5p - CDKN1A ceRNA regulatory axis associated with IS. Our findings provide new insights into the pathogenesis of IS and offer promising diagnostic and predictive biomarkers.

## 1 Introduction

Stroke is the leading cause of permanent disability (Diener and Hankey, 2020). The latest global burden of disease (GBD) study (GBD 2019 Diseases and Injuries Collaborators, 2020) indicated that stroke is the leading cause of death in the Chinese population. The overall lifetime risk of stroke in China was 39.9%, ranking first worldwide. Ischemic stroke (IS) is one of the two major subtypes of strokes, accounting for approximately 80% of all subtypes of strokes (Wang et al., 2017), and is the focus of stroke prevention and treatment. At present, stroke diagnosis mainly relies on clinical symptoms and medical imaging technology. Thrombolytic therapy is the most effective treatment for acute IS (AIS). However, the time window for therapy is narrow, and most patients do not exhibit typical imaging changes in time. Missing this therapeutic window may lead to irreversible brain damage (Herpich and Rincon, 2020; Phipps and Cronin, 2020). Therefore, the emergence of biomarkers for the diagnosis and prediction of IS has come to fruition.

These biomarkers include numerous IS susceptibility genes, such as matrix metalloproteinase-9 (MMP-9) (Singh et al., 2018; Lorente et al., 2019), C-reactive protein (CRP) (Kitagawa et al., 2017), and metabolic intermediate plasma homocysteine (Hcy) (Chen et al., 2017). However, protein-coding regions represent only 1.5%–2% of the human genome, and the non-protein-coding portion of the genome is of crucial functional importance for normal development and physiology, as well as diseases (Esteller, 2011). MiRNAs, one kind of non-conding RNA (ncRNA), have been reported that play crucial role in IS. MiR-145, miR-424, and miR-223 were differentially expressed in patients with is (Zhu et al., 2014). Previous studies have explained that there were many miRNAs involved in the post-transcriptional regulation of Nrf2, and 85 miRNAs can bind to cytoplasmic Nrf2 and affect its translation (Papp et al., 2012). MiR-93 inhibited the expression of Nrf2 and heme oxygenase-1 (HO-1) by targeting Nrf2 (Wang et al., 2016). The Nrf2/HO-1 pathway was an important cellular defense mechanism against oxidative stress induced by ischemia/reperfusion (Jiang et al., 2017). Circular RNAs (circRNAs), another kind of ncRNA, have unique covalently-closed structures, which are insensitive to nucleic acid exonucleases and confers relative higher pharmaceutical stability compared to other linear RNAs, such as miRNAs and mRNAs. Additionally, circRNAs are conserved across species. Therefore, circRNAs have obvious advantages as clinical diagnostic biomarkers. CircRNAs have been reported to play important role in the physiology and pathology of IS. A study found that the expression of circFUNDC1 (hsa_circ_0007290) increased in patients with AIS, and it combined with other circRNAs, with a specificity of 91% and a sensitivity of 71.5% in the diagnosis of AIS (Zuo et al., 2020). Meanwhile, Bai et al. found that the expression level of circFUNDC1 was increased in serum exosomes of patients with AIS (Bai et al., 2022).

CircRNAs often act as miRNA sponges to regulate gene expression, and circRNA-miRNA-mRNA can be called ceRNA network. The ceRNA network has garnered significant attention in the academic community in recent years, representing a novel paradigm of gene expression regulation. In comparison to the regulatory networks involving individual RNA molecules, the ceRNA regulatory network is characterized by its intricacy and complexity. The ceRNA network establishes connections between the gene expression modulation of mRNAs and non-coding RNAs such as circRNAs, miRNAs, and lncRNAs, thereby facilitating a more profound and comprehensive understanding of the roles played by non-coding RNAs in critical biological phenomena, including cellular development and the molecular mechanisms underlying diseases. Among them, the ceRNA mechanism of IS-associated circRNAs has been extensively investigated. The ceRNA network guided by circRNAs typically satisfies the following conditions: circRNAs can act as “sponges” for endogenous miRNAs by possessing miRNA response elements or binding sites, thereby regulating the interaction between miRNAs and their target mRNAs (Kristensen et al., 2019). Thus, circRNAs can regulate mRNA expression and protein levels through ceRNA networks. These networks contain clues to the pathogenesis of IS, and the circRNA-related ceRNA networks associated with IS may serve as potential therapeutic targets. For example, circHECTD1 was overexpressed in plasma samples from mouse stroke models of transient middle cerebral artery occlusion (tMCAO) and patients with AIS. CircHECTD1 overexpression increased the risk of IS via miR-142/TIPARP (Han et al., 2018). In addition, Chen et al. found that circUCK2 was involved in IS regulation through the circUCK2/miR-125b-5p/GDF11 functional network (Chen et al., 2020a). Another study found that circSHOC2 regulated autophagy and acted on the miR-7670-3p/SIRT1 axis (Chen et al., 2020b), thereby inhibiting neuronal apoptosis and reducing neuronal damage.

**FIGURE 1:**
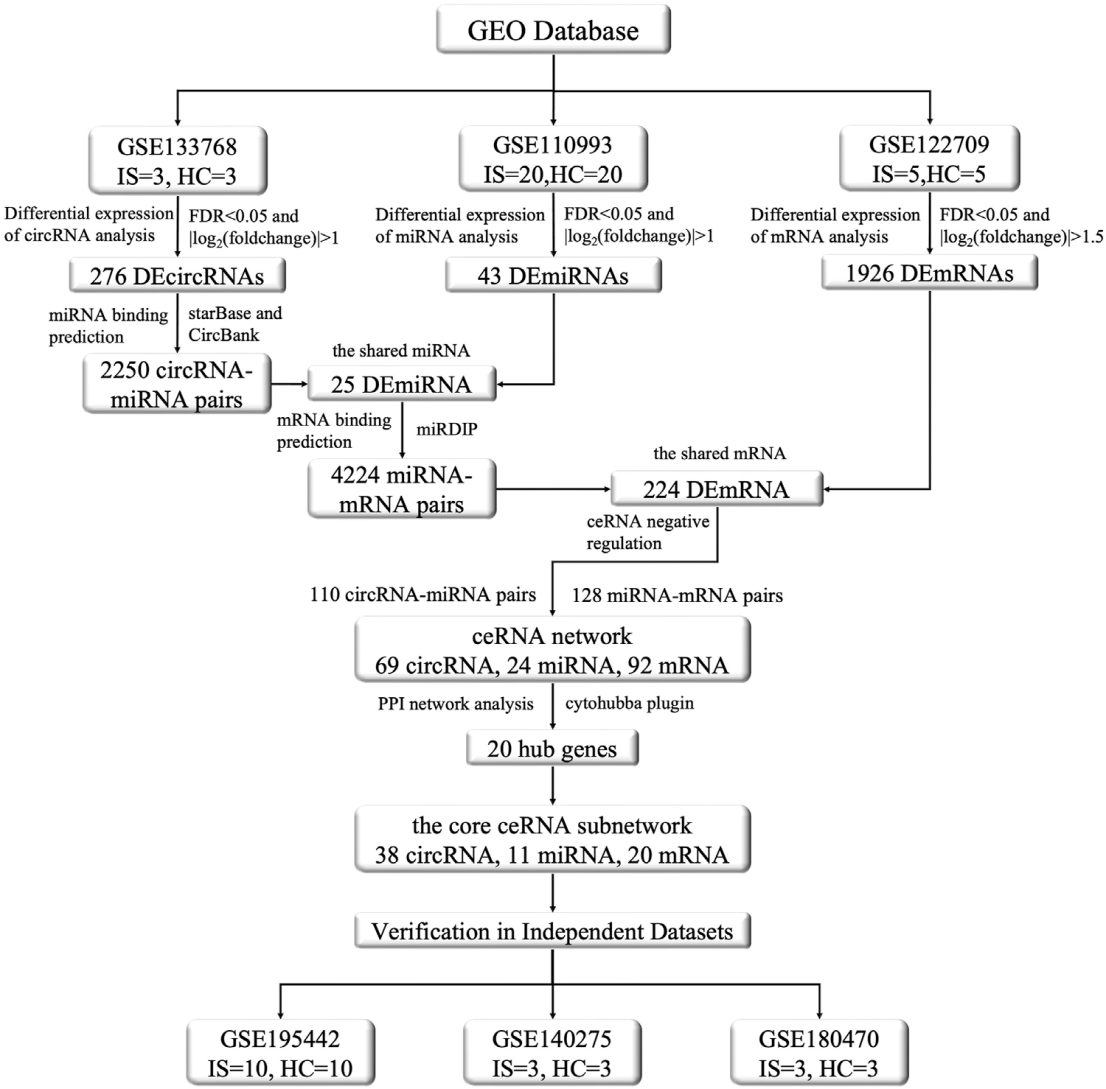
The flow chart of this study. This study contains the following three main aspects: (1) Dowloading and pre-processing datasets. The datasets included circRNA microarray dataset (GSE133768), miRNA and mRNA sequencing dataset (GSE110993, GSE122709). Differential expression analysis, prediction of miRNAs targeted by DEcircRNAs, prediction of DEmiRNAs binding mRNAs, gene enrichment analysis, etc., were conducted on the aforementioned datasets to explore DEcircRNAs, DEmiRNAs, and DEmRNAs and their functional roles between IS and normal control groups. (2) Construction of ceRNA regulatory network and selection of core genes. By integrating the DEcircRNAs, DEmiRNAs, and DEmRNAs obtained in the first step and screening their interactions, we constructed the ceRNA functional network of circRNA-miRNA-mRNA and visualized it using the Cytoscape software. We used the STRING protein database to construct a protein interaction network and used the MCODE algorithm in cytohubba to screen the core genes, and then selected the core sub-network of the ceRNA network based on them. (3) Validation by other independent datasets. For the nodes involved in the above core sub-network, we used other datasets to verify their significance and consistency in differential expression. The validation set were GSE195442 (circRNA), GSE199942 (miRNA), GSE140275 and GSE180470 (mRNA).

The above studies employed sequencing analyses of individual circRNAs and predictions of target miRNAs and mRNAs, and their results were possibly subject to a certain degree of artificial bias. To address this point, we screened the circRNA, miRNA, and mRNA expression profiles of patients through the National Center for Biotechnology Information Gene Expression Omnibus (GEO) database via integrated analysis, constructed an IS-associated ceRNA network which included 69 circRNAs, 24 miRNAs, and 92 mRNAs. We analyzed and validated the core sub-network of the ceRNA network to obtain promising diagnostic biomarkers for IS. This network will facilitate the identification of new treatment targets. The flow chart illustrating the steps of the whole analysis was shown in Figure 1.

## 2 Materials and methods

### 2.1 Data preparation

We downloaded circRNA, miRNA, and mRNA expression profile datasets from the public GEO database (http://www.ncbi.nlm.nih.gov/geo/). The circRNA data were obtained from GSE133768, which included data from plasma samples collected within 72 h of symptom onset from 3 IS patients and 3 healthy controls (HCs). This data was sequenced on the GPL21825 platform using Agilent Arraystar Human CircRNA microarray V2, and 10,799 probes were yielded. MiRNA data were downloaded from GSE110993, which included data from samples collected from 20 patients with IS and 20 HCs. Platelet-poor plasma samples from these patients were collected within 24 h of symptom onset. High-throughput transcriptome sequencing, performed on the GPL15456 platform using Illumina HiScanSQ, yielded 4,447 probes. GSE122709 contained mRNA and lncRNA data from 5 IS patients and 5 HCs. Samples of peripheral blood mononuclear cells were collected from patients 24 h and 7 days after the onset of symptoms. High-throughput transcriptome sequencing was performed on the GPL20795 platform using HiSeq X Ten, and 54,262 probes were yielded.

### 2.2 Differential expression analysis of circRNAs

After the circRNA microarray data were downloaded, an expression matrix containing the 6-digit circRNA_IDs for 10,798 circRNAs was obtained using Perl script conversion, based on the GPL21825 platform annotation information. Then, we used the “limma” package (version 3.52.2, https://bioconductor.org/packages/) in R software (version 4.0.3, https://www.r-project.org/) to identify differentially expressed circRNAs (DEcircRNAs), with FDR <0.05 and |log2FC| > 1 as screening criteria. In addition, to facilitate miRNA prediction, we transformed the 6-digit circRNA_IDs to 7-digit circRNA_IDs using the ID relationship conversion file from the CircBase database (http://www.circbase.org/).

### 2.3 Differential expression analysis of miRNAs

After downloading the miRNA sequencing expression matrix, we removed im-mature miRNAs starting with “hsa-mir” and obtained an expression matrix comprising 2,587 mature miRNAs. We used the “DESeq2” package (version 1.34.0, https://bioconductor.org/packages/release/bioc/html/DESeq2.html) to identify differentially expressed miRNAs (DEmiRNAs), using FDR <0.05 and |log2FC| > 1 as screening criteria.

### 2.4 Differential expression analysis of mRNAs

MRNA expression data were downloaded from GSE122709, including 5 HCs and 5 IS patients within 24 h of the onset of symptoms. To exclude the lncRNA data from the expression matrix, *Homo sapiens*. GRCh38 was used as the reference genome, which is available from Ensembl (https://asia.ensembl.org/index.html/).

For more accurate results, we both used “DESeq2” and “EdgeR” (version 3.36.0, https://bioconductor.org/packages/release/bioc/html/edgeR.html) packages to identify differentially expressed mRNAs (DEmRNAs). The DEmRNAs were then screened, using FDR <0.05 and |log2FC| > 1.5 as criteria. The final DEmRNAs were the shared DEmRNAs using “DESeq2” and “EdgeR” packages.

### 2.5 Functional enrichment analysis

To reveal the functions of the DEmRNAs, we performed Gene Ontology (GO) and Kyoto Encyclopedia of Genes and Genomes (KEGG) pathway analyses using R software (Kanehisa and Goto, 2000; Gene Ontology Consortium, 2015). The Entrez ID for each DEmRNA was obtained using the R package “org.Hs.e.g.,.db.” (version, 3.14.0, https://bioconductor.org/packages/release/data/annottion/html/org.Hs.eg.db.html). To elucidate the mechanisms underlying the association of DEmRNAs with biological processes (BPs), GO and KEGG function annotations were analyzed using “ggplot2” (version 3.3.5, https://github.com/tidyverse/ggplot2), “enrichplot” (version 1.14, https://bioconductor.org/packages/release/bioc/html/enrichplot.html), and “clusterProfiler” (version 4.2.2, https://bioconductor.org/packages/release/bioc/html/clusterProfiler.html) packages.

### 2.6 Construction of the ceRNA network

**FIGURE 2:**
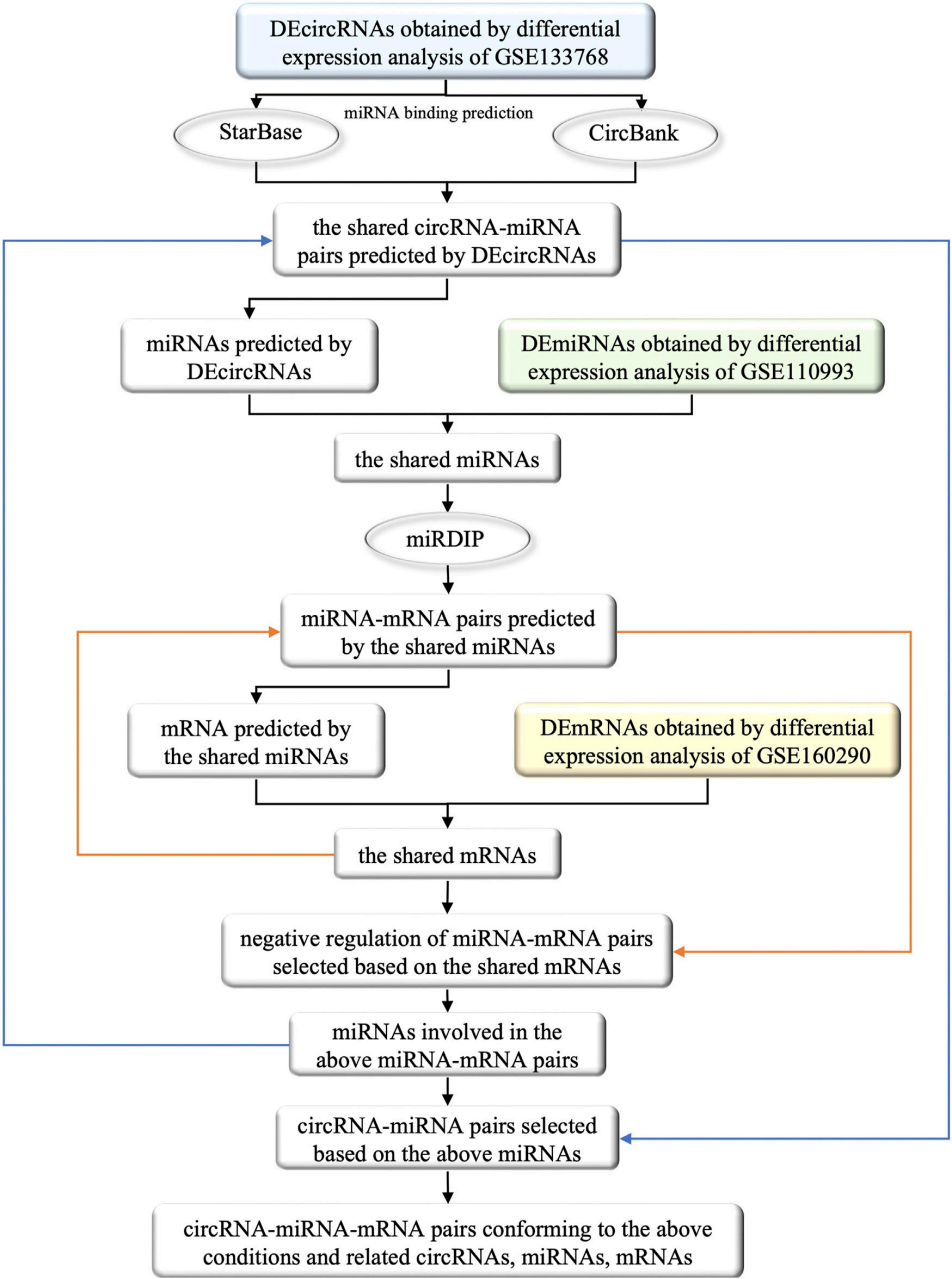
The flow chart of the selection of circRNAs, miRNAs and mRNAs involved in the ceRNA network and their interrelationships. The square boxes indicate the screened data and the oval boxes indicate the databases that were used. In particular, blue, green and yellow boxes indicate DEcircRNAs, DEmiRNAs and DEmRNAs obtained from differential expression analysis of circRNAs, miRNAs and mRNAs that were extracted from GEO database, respectively. The orange arrow demonstrates the process of incorporating the shared mRNAs into the previously identified miRNA-mRNA pairs, and filtering out the negative regulatory miRNA-mRNA pairs associated with these mRNAs. Similarly, the blue arrow illustrates the process of obtaining miRNA from the screened miRNA-mRNA pairs in the previous step, and incorporating these miRNAs into previously identified circRNA-miRNA pairs to obtain the circRNA-miRNA pairs involved in these miRNAs. Ultimately, by identifying the miRNAs that satisfies both the newly obtained circRNA-miRNA pairs and miRNA-mRNA pairs, we successfully constructed a ceRNA functional network of circRNA-miRNA-mRNA.

In order to facilitate understanding, the specific process of the construction of ceRNA network was shown in Figure 2. Initially, we utilized two databases, namely, StarBase (v2.0, https://starbase.sysu.edu.cn/) and CircBank (http://www.circbank.cn/), to forecast the miRNA binding sites of DEcircRNAs. The specific steps involved in this process are as follows: 1) Due to the unavailability of batch prediction of miRNA binding sites and the non-uniqueness of the circRNA naming convention in the files containing circRNA-miRNA pairs downloaded directly from StarBase, it was not feasible to conduct subsequent batch predictions. Therefore, we utilized R software to batch download all circRNA-miRNA pairs corresponding to circRNA IDs named with 7 digits. After removing duplicates, we merged the resulting 3,502,309 circRNA-miRNA pairs and filtered out those corresponding to DEcircRNAs. 2) From the download section of CircBank, we obtained a file containing all circRNA-miRNA pairs, which comprised a total of 16,844,374 pairs. Similarly, we utilized R software to filter out the circRNA-miRNA pairs corresponding to DEcircRNAs. 3) The predicted circRNA-miRNA pairs from both StarBase and CircBank were intersected, and duplicates were removed to obtain the shared predicted miRNAs for DEcircRNAs.

We further utilized the mirDIP database (http://ophid.utoronto.ca/mirDIP/index.jsp) to predict the mRNA binding sites of the miRNAs predicted by DEcircRNAs. Specifically, we took the intersection of DEcircRNA-predicted miRNAs and DEmiRNAs, inputted the shared miRNAs into the “miRNAs” column of mirDIP, selected the corresponding “Score class” as “Top 1%", obtained miRNA-mRNA pairs, and removed duplicates to obtain the shared miRNA-predicted mRNAs.

We then took the intersection of the predicted mRNAs and DEmRNAs, examined the miRNA-mRNA pairs involved in the shared mRNAs, selected negative regulatory miRNA-mRNA pairs (Only the case of miRNA-mRNA pairs with negative regulation is discussed in this study), and obtained corresponding miRNAs and mRNAs. We further obtained the circRNA-miRNA pairs in which the above miRNAs participated, and eliminated the miRNAs that were not correlated with circRNAs.Through the remaining miRNAs and their participating circRNA-miRNA pairs, miRNA-mRNA pairs, we obtained the selected circRNAs, miRNAs and mRNAs to construct the ceRNA network.

Based on the above analysis, we constructed a ceRNA functional network composed of circRNA-miRNA-mRNA pairs. For visualization, we used Cytoscape v3.9.0 (https://cytoscape.org/) to map the ceRNA functional network.

### 2.7 Integration of protein-protein interaction (PPI) networks and construction of core sub-network

To identify hub genes, we performed PPI network analysis of all mRNAs involved in the ceRNA network. We searched for interactions between genes using the STRING database (http://stringdb.org/), with an interaction score threshold of "> 0.40". To construct the core sub-network of the ceRNA network, we visualized the PPI network using Cytoscape and selected hub genes using the Cytohubba plugin.

### 2.8 Validation in independent datasets

To minimize bias, we utilized multiple independent datasets to validate the circRNAs, miRNAs, and mRNAs which contained in the core sub-network. GSE195442, which included microarray data from plasma exosome samples collected from 10 patients with IS and 10 HCs, was used as the validation dataset for circRNAs. GSE199942, which included sequencing data from serum samples collected from 5 IS patients and 5 HCs, was used as the validation dataset for miRNAs. GSE180470 and GSE140275 were used as mRNA validation datasets, both of the datasets containing sequencing data from blood samples collected from 3 IS patients and 3 HCs. To verify the accuracy of the results, we used the same processing methods and analysis criteria when constructing the ceRNA network.

## 3 Results

### 3.1 Identification of DEcircRNAs

**FIGURE 3:**
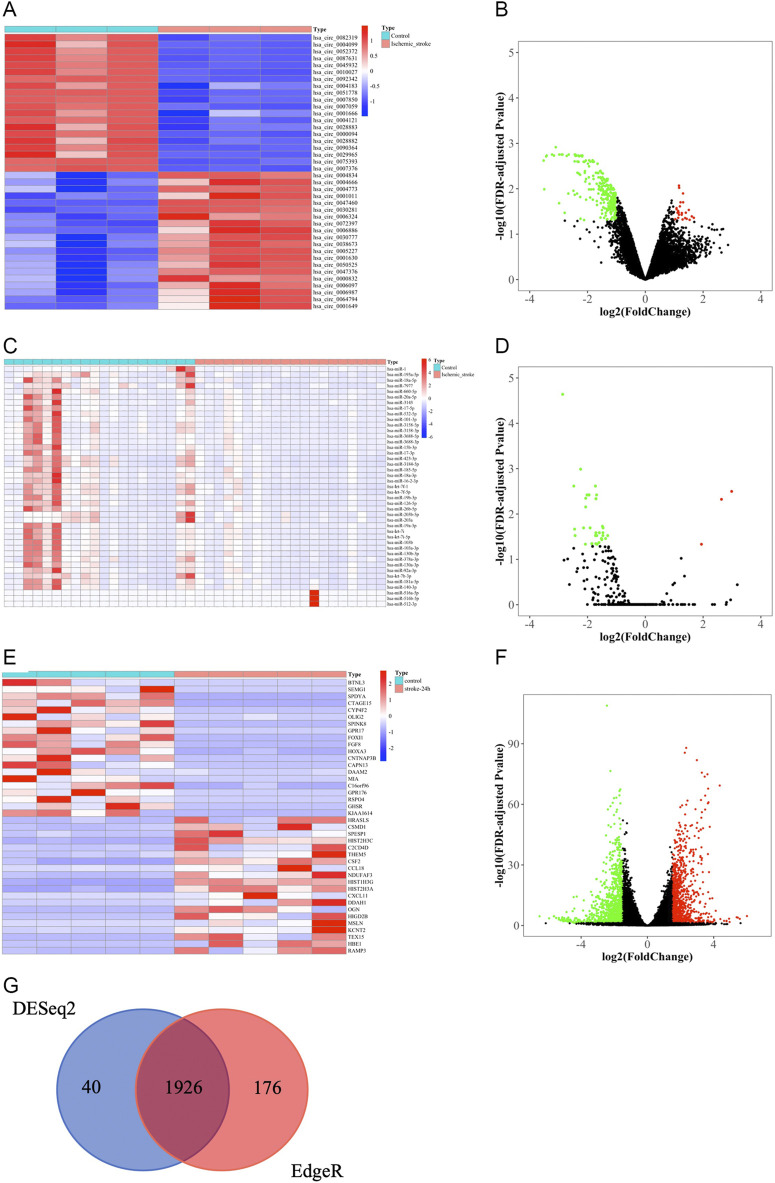
Identification of DEcircRNAs, DEmiRNAs, and DEmRNAs. **(A)** The heat map of top 20 upregulated and top 20 downregulated DEcircRNAs. **(B)** Volcano plot of circRNAs. Green and red represent downregulated and upregulated DEcircRNAs, respectively. |log2FC| > 1, FDR < 0.05. circRNAs, circular RNAs. **(C)** The heat map of all DEmiRNAs. **(D)** Volcano plot of miRNAs. Green and red represent downregulated and upregulated DEmiRNAs, respectively. |log2FC|> 1, FDR < 0.05. miRNAs, microRNAs. **(E)** The heatmap of top 20 upregulated and downregulated DEmRNAs based on “DESeq2” package. **(F)** Volcano plot of mRNAs that were screened based on “DESeq2” package. Green and red represent downregulated and upregulated DEmRNAs, respectively. |log2FC| > 1.5, FDR < 0.05. mRNAs, messenger RNAs. **(G)** Venn diagram of the shared DEmRNAs obtained by “DESeq2” and “EdgeR” packages.

We obtained 276 DEcircRNAs, each with a 7-digit circRNA_ID, by analyzing the expression profiles from GSE133768. Among them, 24 upregulated and 252 downregulated DEcircRNAs were associated with IS. We then plotted the top 20 upregulated and top 20 downregulated DEcircRNAs using heat maps and volcano plots (Figures 3A,B). The basic information for DEcircRNAs were shown in Table1.

**TABLE 1:** The top 20 upregulated and 20 downregulated DEcircRNAs.

Expression	circRNA	log2FC	*p*-value	FDR
Upregulated	hsa_circ_0001649	1.679235	0.001848	0.043638
	hsa_circ_0064794	1.608807	0.001626	0.040557
	hsa_circ_0006987	1.533734	0.002287	0.048381
	hsa_circ_0006097	1.504968	0.000935	0.029290
	hsa_circ_0000832	1.432463	0.002049	0.045897
	hsa_circ_0047376	1.401033	0.001176	0.033688
	hsa_circ_0050525	1.377491	0.001917	0.044145
	hsa_circ_0001630	1.308806	0.000447	0.019868
	hsa_circ_0005227	1.305516	0.000193	0.012681
	hsa_circ_0038673	1.290057	0.002223	0.047440
	hsa_circ_0030777	1.264306	0.001249	0.034217
	hsa_circ_0006886	1.259983	0.001403	0.037324
	hsa_circ_0072397	1.217144	0.000451	0.019868
	hsa_circ_0006324	1.196506	0.000644	0.024402
	hsa_circ_0030281	1.170937	0.000100	0.009525
	hsa_circ_0047460	1.164586	0.000083	0.008525
	hsa_circ_0001011	1.164247	0.001409	0.037362
	hsa_circ_0004773	1.151203	0.001163	0.033545
	hsa_circ_0004666	1.139829	0.001852	0.043638
	hsa_circ_0004834	1.136592	0.001012	0.030868
Downregulated	hsa_circ_0007376	−2.423281	0.000001	0.001764
	hsa_circ_0075393	−2.440287	0.000002	0.001764
	hsa_circ_0029965	−2.442756	0.000236	0.013586
	hsa_circ_0090364	−2.445093	0.000007	0.002498
	hsa_circ_0028882	−2.487405	0.000150	0.011085
	hsa_circ_0000094	−2.651994	0.000001	0.001764
	hsa_circ_0028883	−2.664252	0.000203	0.012745
	hsa_circ_0004121	−2.720927	0.000004	0.001890
	hsa_circ_0001666	−2.790803	0.001214	0.033881
	hsa_circ_0007059	−2.865738	0.000001	0.001764
	hsa_circ_0007850	−2.894363	0.000002	0.001764
	hsa_circ_0051778	−2.978394	0.000001	0.001764
	hsa_circ_0004183	−2.984381	0.000509	0.020814
	hsa_circ_0092342	−3.100302	0.000000	0.001216
	hsa_circ_0010027	−3.161598	0.000003	0.001861
	hsa_circ_0045932	−3.229383	0.000002	0.001764
	hsa_circ_0087631	−3.413685	0.000003	0.001861
	hsa_circ_0052372	−3.431524	0.000004	0.001943
	hsa_circ_0004099	−3.497811	0.000115	0.010272
	hsa_circ_0082319	−3.519317	0.000006	0.002395

### 3.2 Identification of DEmiRNAs

Due to the limited number of mature miRNAs obtained by sequencing, we used “DESeq2” package in R software to analyze the GSE110993 expression profiles. We obtained 43 DEmiRNAs, of which 3 were upregulated and 40 were downregulated (Table 2; Figures 3C,D).

**TABLE 2:** The total of 43 DEmiRNAs.

Expression	miRNA	log2FC	*p*-value	FDR
Upregulated	hsa-miR-516b-5p	2.635913	0.000077	0.004739
	hsa-miR-516a-5p	1.943530	0.002309	0.046404
	hsa-miR-512-3p	2.987824	0.000022	0.003170
Downregulated	hsa-miR-92a-3p	−1.466306	0.000690	0.025814
	hsa-miR-7977	−2.430273	0.001064	0.029846
	hsa-miR-660-5p	−2.239786	0.000002	0.001026
	hsa-miR-532-5p	−2.063253	0.000057	0.004500
	hsa-miR-423-3p	−1.726925	0.000014	0.002414
	hsa-miR-378a-3p	−1.483360	0.000346	0.018673
	hsa-miR-3688-5p	−1.910698	0.000444	0.020202
	hsa-miR-3688-3p	−1.910698	0.000444	0.020202
	hsa-miR-3184-5p	−1.726925	0.000014	0.002414
	hsa-miR-3158-5p	−1.946975	0.000044	0.003793
	hsa-miR-3158-3p	−1.946975	0.000044	0.003793
	hsa-miR-3143	−2.074553	0.002173	0.046329
	hsa-miR-26b-5p	−1.678483	0.000997	0.029846
	hsa-miR-20a-5p	−2.136953	0.000438	0.020202
	hsa-miR-203b-5p	−1.668656	0.002252	0.046329
	hsa-miR-203a	−1.668656	0.002252	0.046329
	hsa-miR-19b-3p	−1.695119	0.000988	0.029846
	hsa-miR-19a-3p	−1.604727	0.001776	0.042616
	hsa-miR-193a-5p	−2.475112	0.000013	0.002414
	hsa-miR-18a-5p	−2.447728	0.000523	0.021520
	hsa-miR-18a-3p	−1.715550	0.002033	0.045030
	hsa-miR-185-5p	−1.726137	0.001071	0.029846
	hsa-miR-181a-5p	−1.375532	0.001312	0.034726
	hsa-miR-17-5p	−2.064623	0.000121	0.006977
	hsa-miR-17-3p	−1.844967	0.001923	0.044894
	hsa-miR-16-2-3p	−1.711612	0.000743	0.025814
	hsa-miR-15b-3p	−1.853834	0.002021	0.045030
	hsa-miR-140-3p	−1.301397	0.001033	0.029846
	hsa-miR-130b-3p	−1.505549	0.001489	0.036754
	hsa-miR-130a-3p	−1.467966	0.000982	0.029846
	hsa-miR-126-5p	−1.686944	0.000039	0.003793
	hsa-miR-103b	−1.519878	0.001367	0.034726
	hsa-miR-103a-3p	−1.519878	0.001367	0.034726
	hsa-miR-101-3p	−2.015893	0.000040	0.003793
	hsa-miR-1	−2.861548	0.000000	0.000023
	hsa-let-7i-5p	−1.591290	0.000747	0.025814
	hsa-let-7i	−1.591497	0.000746	0.025814
	hsa-let-7f-5p	−1.708476	0.000069	0.004608
	hsa-let-7f-1	−1.709172	0.000069	0.004608
	hsa-let-7b-3p	−1.462872	0.000468	0.020232

### 3.3 Identification of DEmRNAs and function enrichment analysis

We screened the expression matrix for 19,298 mRNAs from the GSE122709 dataset. Differention expressing analysis found 1966 DEmRNAs (985 upregulated and 981 downregulated) using the “DESeq2” package. We then plotted the top 20 upregulated and top 20 downregulated DEmRNAs using heat maps and volcano plots (Table 3; Figures 3E,F). We used “EdgeR” package to obtain more accurate differential analysis results and identified 2,102 DEmRNAs, of which 1,090 were upregulated and 1,012 were downregulated; heat maps and volcano plots are presented in the Supplementary Materials (Supplementary Table S1; Supplementary Figure S1A, B). The results of the two analyses were intersected, yielding 1926 DEmRNAs (Figure 3G).

**TABLE 3:** The top 20 upregulated and 20 downregulated DEmRNAs.

Expression	mRNA	log2FC	*p*-value	FDR
Upregulated	RAMP3	6.005372	0.000005	0.000021
	HBE1	5.571703	0.000144	0.000493
	TEX15	5.452020	0.000033	0.000127
	KCNT2	5.385740	0.000021	0.000083
	MSLN	4.956401	0.007196	0.017393
	HIGD2B	4.926683	0.000120	0.000418
	OGN	4.774183	0.001611	0.004471
	DDAH1	4.773587	0.003045	0.007982
	CXCL11	4.624720	0.001732	0.004780
	HIST2H3A	4.401545	0.000188	0.000632
	HIST1H3G	4.362263	0.000000	0.000000
	NDUFAF3	4.122088	0.000000	0.000000
	CCL18	4.121776	0.021198	0.045247
	CSF2	4.040330	0.001014	0.002949
	THEM5	4.021911	0.000291	0.000942
	C2CD4D	3.956950	0.005955	0.014646
	HIST2H3C	3.917556	0.000000	0.000000
	SPESP1	3.892726	0.008030	0.019164
	CSMD1	3.853778	0.000009	0.000038
	HRASLS	3.849179	0.013554	0.030617
Downregulated	KIAA1614	−4.922627	0.001801	0.004951
	GHSR	−4.927454	0.001071	0.003093
	RSPO4	−4.948212	0.001258	0.003575
	GPR176	−4.967716	0.000907	0.002660
	C16orf96	−5.013368	0.000063	0.000231
	MIA	−5.051163	0.003078	0.008056
	DAAM2	−5.063865	0.000662	0.001988
	CAPN13	−5.088329	0.002151	0.005819
	CNTNAP3B	−5.105684	0.001370	0.003860
	HOXA3	−5.135764	0.000023	0.000090
	FGF8	−5.142336	0.000181	0.000609
	FOXI1	−5.158171	0.000177	0.000596
	GPR17	−5.235161	0.000927	0.002714
	SPINK8	−5.340008	0.000049	0.000181
	OLIG2	−5.344356	0.000282	0.000914
	CYP4F2	−5.503473	0.000048	0.000179
	CTAGE15	−5.626503	0.000006	0.000026
	SPDYA	−5.645933	0.000006	0.000027
	SEMG1	−5.927450	0.000079	0.000286
	BTNL3	−6.509618	0.000007	0.000028

**FIGURE 4:**
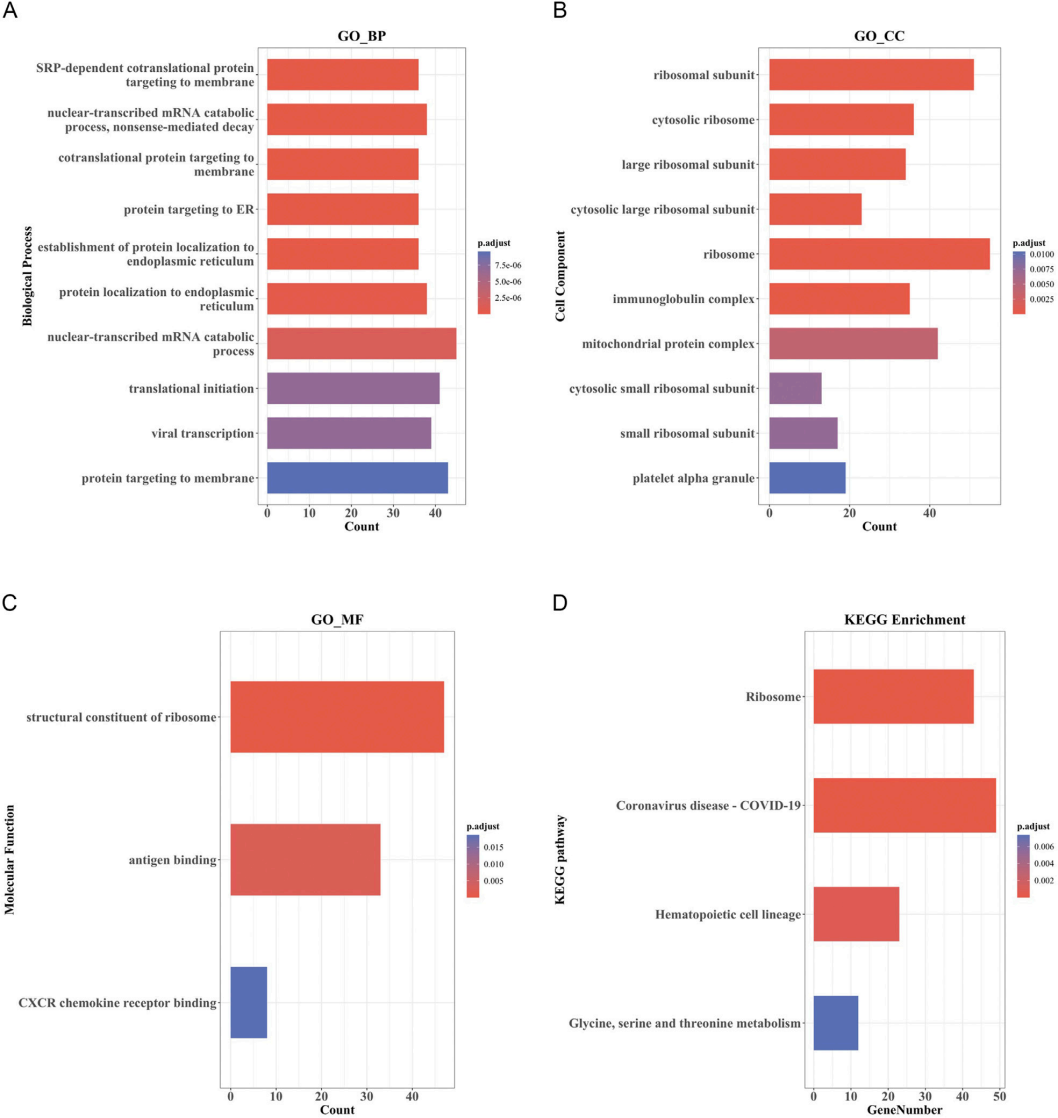
GO and KEGG pathway enrichment analysis of DEmRNAs. **(A)** Bar Plot of BP. **(B)** Bar Plot of CC. **(C)** Bar Plot of MF. **(D)** Bar Plot of KEGG. (GO, Gene Ontology; BP, biological processes; CC, cell component; MF, molecular function).

The top 10 results of GO enrichment analysis indicated that the DEmRNAs were mainly involved in transcription, translation, nuclear-transcribed mRNA catabolism, and other related BPs (Figure 4A). For the cellular components, DEmRNAs were mainly enriched in the large and small ribosome subunits and mitochondrial protein complexes (Figure 4B). In the context of molecular functions, the DEmRNAs were significantly involved in the structural constituents of the ribosome, CXCR chemokine receptor binding, and c antigen binding (Figure 4C). Furthermore, KEGG pathway analysis showed that the DEmRNAs were involved in the ribosome; hematopoietic cell lineage; glycine, serine, and threonine metabolism; and coronavirus disease-COVID-19 pathways (Figure 4D).

### 3.4 Construction of ceRNA regulatory network

**FIGURE 5:**
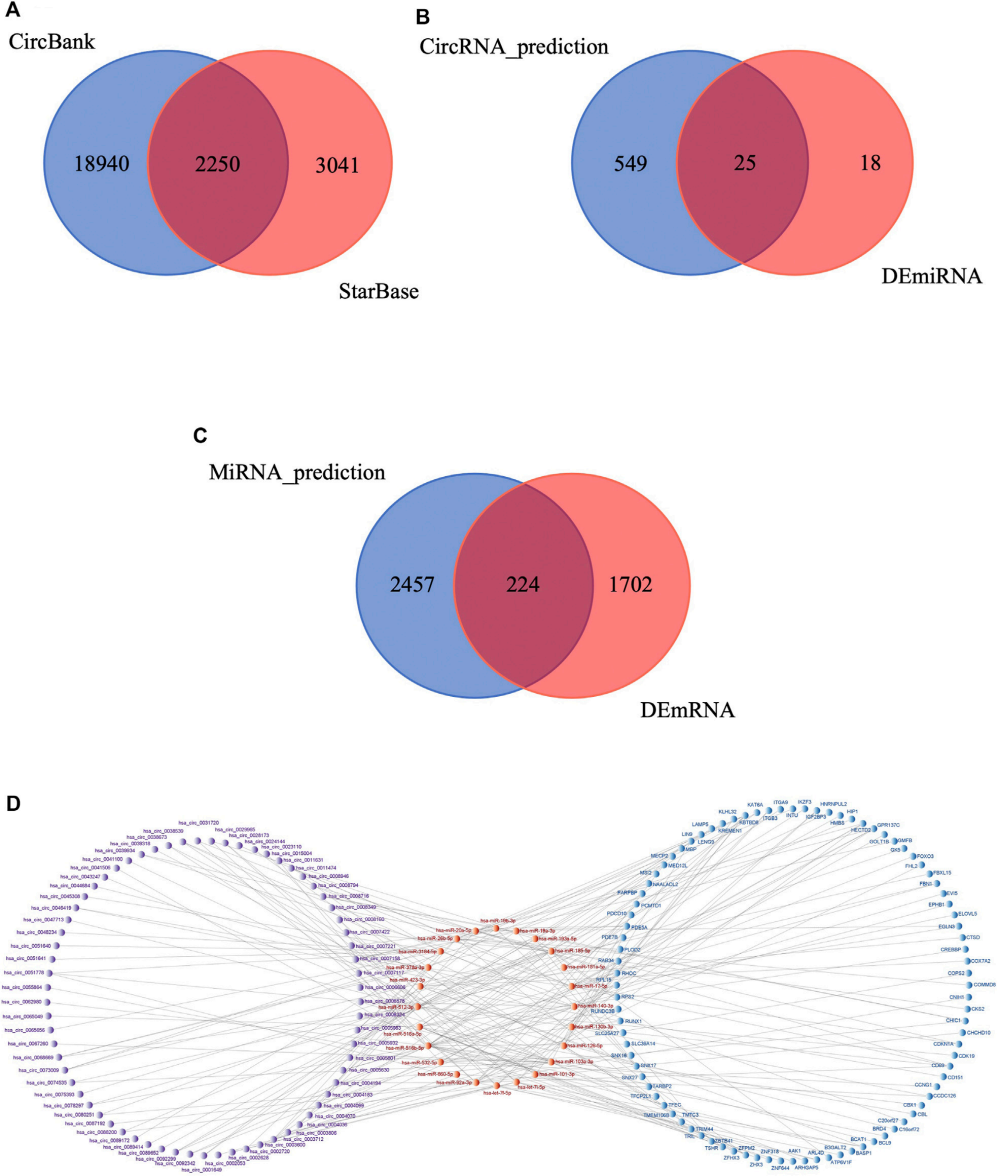
Construction of circRNA-associated ceRNA network in IS. **(A)** Venn diagram of the shared circRNA-miRNA pairs predicted from CircBank and StarBase databases for 276 DEcircRNAs. **(B)** Venn diagram of 574 miRNAs predicted by DEcircRNAs and 43 DEmiRNAs. **(C)** Venn diagram of 2,681 mRNAs predicted by DEmiRNAs and 1926 DEmRNAs. **(D)** Visualization of the ceRNA network. The red nodes represent miRNAs, the purple nodes represent circRNAs, the blue nodes represent mRNAs.

First, StarBase and CircBank databases were used to predict the target miRNAs of the 276 DEcircRNAs; 5,291 and 21,190 circRNA-miRNA pairs were predicted, respectively. The results were intersected to obtain 2,250 circRNA-miRNA pairs (Figure 5A), comprising 221 circRNAs and 574 miRNAs. We then intersected these 574 predicted miRNAs with the 43 DEmiRNAs to obtain 25 miRNAs (Figure 5B). We used mirDIP database to predict the target mRNAs of the 25 miRNAs, resulting in 4,224 miRNA-mRNA pairs involving 2,681 mRNAs. This result was intersected with the 1926 DEmRNAs, yielding 224 mRNAs (Figure 5C). We verified the differential expression of the screened circRNAs, miRNAs, and mRNAs in the GEO datasets and subjected them to further screening according to the regulatory mechanism of ceRNAs. Using Cytoscape software, we successfully built a circRNA-based ceRNA regulatory network (Figure 5D) comprising 110 circRNA-miRNA pairs and 128 miRNA-mRNA pairs that included 69 circRNAs, 24 miRNAs, and 92 mRNAs.

### 3.5 PPI network analysis and core ceRNA sub-network construction

**FIGURE 6:**
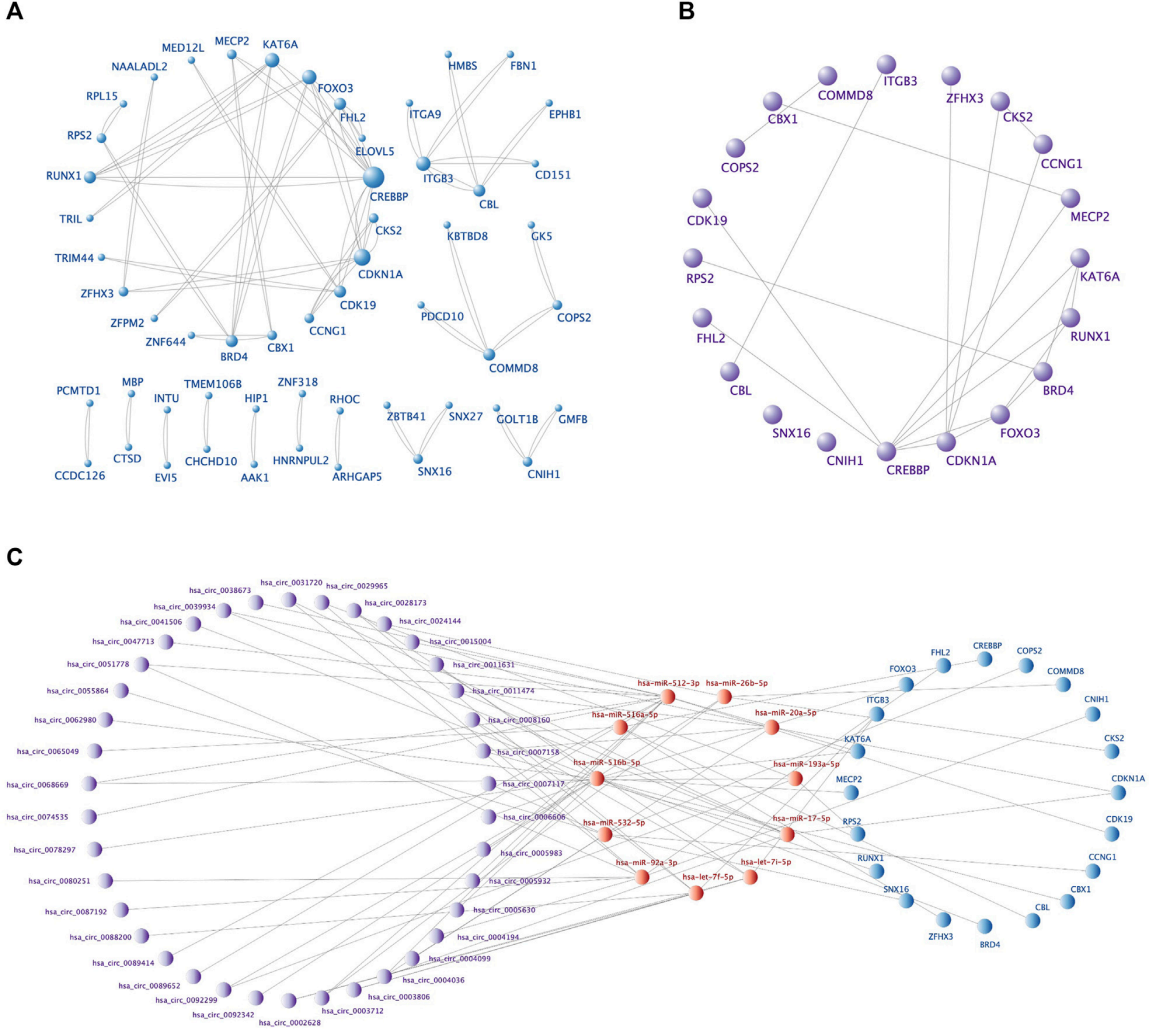
The PPI network, top 20 hub genes, and core ceRNA sub-network. **(A)** The PPI network of 92 DEmRNAs constructed by Cytoscape software. Larger nodes indicate higher gene connectivity. **(B)** The top 20 hub genes obtained by Cytohubba plugin. **(C)** The core sub-network screened from the ceRNA network by the 20 hub genes.

The PPI network, including 92 nodes and 47 edges, was constructed using the STRING protein database. We used Cytoscape software to visualize the PPI network (Figure 6A) and filtered the top 20 hub genes using the Cytohubba plugin (Table 4; Figure 6B). We then screened the corresponding sub-network from the ceRNA network (Figure 6C). The core sub-network included 48 circRNA-miRNA pairs and 23 miRNA-mRNA pairs, with a total of 69 nodes (38 circRNAs, 11 miRNAs, and 20 mRNAs).

**TABLE 4:** Functional information of the top20 genes selected from the PPI network.

Gene	Aliases for genes	Function	Expression
BRD4	HUNK1, HUNKI, MCAP	BRD4 (Bromodomain Containing 4) is a Protein Coding gene. Diseases associated with BRD4 include Cornelia De Lange Syndrome and Cornelia De Lange Syndrome 1. Among its related pathways are 7q11.23 copy number variation syndrome and Chromatin Regulation/Acetylation. Gene Ontology (GO) annotations related to this gene include chromatin binding and lysine-acetylated histone binding. An important paralog of this gene is BRD3	Downregulated
CBL	RNF55, CBL2	CBL (Cbl Proto-Oncogene) is a Protein Coding gene. Diseases associated with CBL include Noonan Syndrome-Like Disorder With Or Without Juvenile Myelomonocytic Leukemia and Juvenile Myelomonocytic Leukemia. Among its related pathways are Negative regulation of FGFR1 signaling and PDGFR-beta signaling pathway. Gene Ontology (GO) annotations related to this gene include DNA-binding transcription factor activity and ligase activity. An important paralog of this gene is CBLB.	Downregulated
CBX1	M31, CBX, HP1Hs-Bet	CBX1 (Chromobox 1) is a Protein Coding gene. Diseases associated with CBX1 include Hyperoxaluria, Primary, Type I and Hutchinson-Gilford Progeria Syndrome. Among its related pathways are HCMV Infection and Chromatin Regulation/Acetylation. Gene Ontology (GO) annotations related to this gene include protein homodimerization activity and chromatin binding. An important paralog of this gene is CBX3	Downregulated
CCNG1	CCNG, Cyclin-G1	CCNG1 (Cyclin G1) is a Protein Coding gene. Diseases associated with CCNG1 include Mantle Cell Lymphoma and Breast Cancer. Among its related pathways are GPCR Pathway and Gene expression (Transcription). Gene Ontology (GO) annotations related to this gene include protein domain specific binding. An important paralog of this gene is CCNG2	Upregulated
CDK19	KIAA1028, CDC2L6, CDK11	CDK19 (Cyclin Dependent Kinase 19) is a Protein Coding gene. Diseases associated with CDK19 include Developmental And Epileptic Encephalopathy 87 and Non-Specific Early-Onset Epileptic Encephalopathy. Among its related pathways are Activation of the pre-replicative complex and PPARA activates gene expression. Gene Ontology (GO) annotations related to this gene include transferase activity, transferring phosphorus-containing groUpregulateds and protein tyrosine kinase activity. An important paralog of this gene is CDK8	Downregulated
CDKN1A	P21, CAP20, CIP1	CDKN1A (Cyclin Dependent Kinase Inhibitor 1A) is a Protein Coding gene. Diseases associated with CDKN1A include Multiple Endocrine Neoplasia, Type I and Tongue Carcinoma. Among its related pathways are Cellular Senescence and Defective binding of RB1 mutants to E2F1, (E2F2, E2F3). Gene Ontology (GO) annotations related to this gene include ubiquitin protein ligase binding and cyclin binding. An important paralog of this gene is CDKN1C	Upregulated
CKS2	Cyclin-Dependent Kinases Regulatory Subunit 2	CKS2 (CDC28 Protein Kinase Regulatory Subunit 2) is a Protein Coding gene. Among its related pathways are Small cell lung cancer. Gene Ontology (GO) annotations related to this gene include cyclin-dependent protein serine/threonine kinase regulator activity. An important paralog of this gene is CKS1B	Upregulated
CNIH1	TGAM77, CNIL, CNIH	CNIH1 (Cornichon Family AMPA Receptor Auxiliary Protein 1) is a Protein Coding gene. Diseases associated with CNIH1 include Variola Major and Schizophrenia. Among its related pathways are Metabolism of proteins and Transport to the Golgi and subsequent modification. An important paralog of this gene is CNIH3	Upregulated
COMMD8	COMM Domain-Containing Protein 8	COMMD8 (COMM Domain Containing 8) is a Protein Coding gene. Among its related pathways are Class I MHC mediated antigen processing and presentation and Metabolism of proteins. An important paralog of this gene is ENSG00000285382	Upregulated
COPS2	TRIP15, CSN2, ALIEN	COPS2 (COP9 Signalosome Subunit 2) is a Protein Coding gene. Diseases associated with COPS2 include Xeroderma Pigmentosum, Complementation GroUpregulated E and Persistent Hyperplastic Primary Vitreous. Among its related pathways are Class I MHC mediated antigen processing and presentation and Metabolism of proteins. Gene Ontology (GO) annotations related to this gene include obsolete signal transducer activity and transcription corepressor activity. An important paralog of this gene is PSMD11	Upregulated
CREBBP	CBP, KAT3A	CREBBP (CREB Binding Protein) is a Protein Coding gene. Diseases associated with CREBBP include Rubinstein-Taybi Syndrome 1 and Menke-Hennekam Syndrome 1. Among its related pathways are Development Ligand-independent activation of ESR1 and ESR2 and MIF Mediated Glucocorticoid Regulation. Gene Ontology (GO) annotations related to this gene include DNA-binding transcription factor activity and transcription factor binding. An important paralog of this gene is EP300	Downregulated
FHL2	SLIM3, DRAL	FHL2 (Four And A Half LIM Domains 2) is a Protein Coding gene. Diseases associated with FHL2 include Familial Isolated Dilated Cardiomyopathy and Rhabdomyosarcoma. Among its related pathways are Ectoderm differentiation and PPARA activates gene expression. Gene Ontology (GO) annotations related to this gene include identical protein binding and transcription coactivator activity. An important paralog of this gene is FHL5	Upregulated
FOXO3	AF6q21, FKHRL1, FOXO3A	FOXO3 (Forkhead Box O3) is a Protein Coding gene. Diseases associated with FOXO3 include Chromosome 6Q Deletion and Aging. Among its related pathways are PI3K/Akt Signaling and PIP3 activates AKT signaling. Gene Ontology (GO) annotations related to this gene include DNA-binding transcription factor activity and protein kinase binding. An important paralog of this gene is FOXO1	Downregulated
ITGB3	GPIIIa, CD61, GP3A	ITGB3 (Integrin Subunit Beta 3) is a Protein Coding gene. Diseases associated with ITGB3 include Bleeding Disorder, Platelet-Type, 24 and Glanzmann Thrombasthenia 2. Among its related pathways are GPCR Pathway and Actin Nucleation by ARP-WASP Complex. Gene Ontology (GO) annotations related to this gene include identical protein binding and protease binding. An important paralog of this gene is ENSG00000259753	Upregulated
KAT6A	MOZ, ZC2HC6A, RUNXBP2	KAT6A (Lysine Acetyltransferase 6A) is a Protein Coding gene. Diseases associated with KAT6A include Arboleda-Tham Syndrome and Syndromic Intellectual Disability. Among its related pathways are Regulation of TP53 Activity through Acetylation and Chromatin organization. Gene Ontology (GO) annotations related to this gene include chromatin binding and transcription coactivator activity. An important paralog of this gene is KAT6B	Downregulated
MECP2	MRX16, MRX79, RTT	MECP2 (Methyl-CpG Binding Protein 2) is a Protein Coding gene. Diseases associated with MECP2 include Rett Syndrome and Encephalopathy, Neonatal Severe, Due To Mecp2 Mutations. Among its related pathways are Ectoderm differentiation and Transcriptional Regulation by MECP2. Gene Ontology (GO) annotations related to this gene include RNA binding and chromatin binding. An important paralog of this gene is MBD4	Downregulated
RPS2	LLREP3, S2	RPS2 (Ribosomal Protein S2) is a Protein Coding gene. Diseases associated with RPS2 include Diamond-Blackfan Anemia. Among its related pathways are Metabolism of proteins and SARS-CoV-2 Infection. Gene Ontology (GO) annotations related to this gene include RNA binding and enzyme binding. An important paralog of this gene is MRPS5	Upregulated
RUNX1	AMLCR1, CBFA2, AML1	RUNX1 (RUNX Family Transcription Factor 1) is a Protein Coding gene. Diseases associated with RUNX1 include Platelet Disorder, Familial, With Associated Myeloid Malignancy and Blood Platelet Disease. Among its related pathways are Gene expression (Transcription) and RUNX1 regulates transcription of genes involved in BCR signaling. Gene Ontology (GO) annotations related to this gene include DNA-binding transcription factor activity and protein homodimerization activity. An important paralog of this gene is RUNX2	Downregulated
SNX16	Sorting Nexin-16	SNX16 (Sorting Nexin 16) is a Protein Coding gene. Diseases associated with SNX16 include Spinocerebellar Ataxia 15. Gene Ontology (GO) annotations related to this gene include identical protein binding and phosphatidylinositol binding. An important paralog of this gene is PXK.	Upregulated
ZFHX3	C16orf47, ZNF927, ATBF1	ZFHX3 (Zinc Finger Homeobox 3) is a Protein Coding gene. Diseases associated with ZFHX3 include Prostate Cancer and Atrial Fibrillation. Among its related pathways are Transcriptional Regulatory Network in Embryonic Stem Cell and Gene expression (Transcription). Gene Ontology (GO) annotations related to this gene include nucleic acid binding and sequence-specific DNA binding. An important paralog of this gene is ZFHX4	Downregulated

### 3.6 Validation of core sub-network in GEO datasets

**FIGURE 7:**
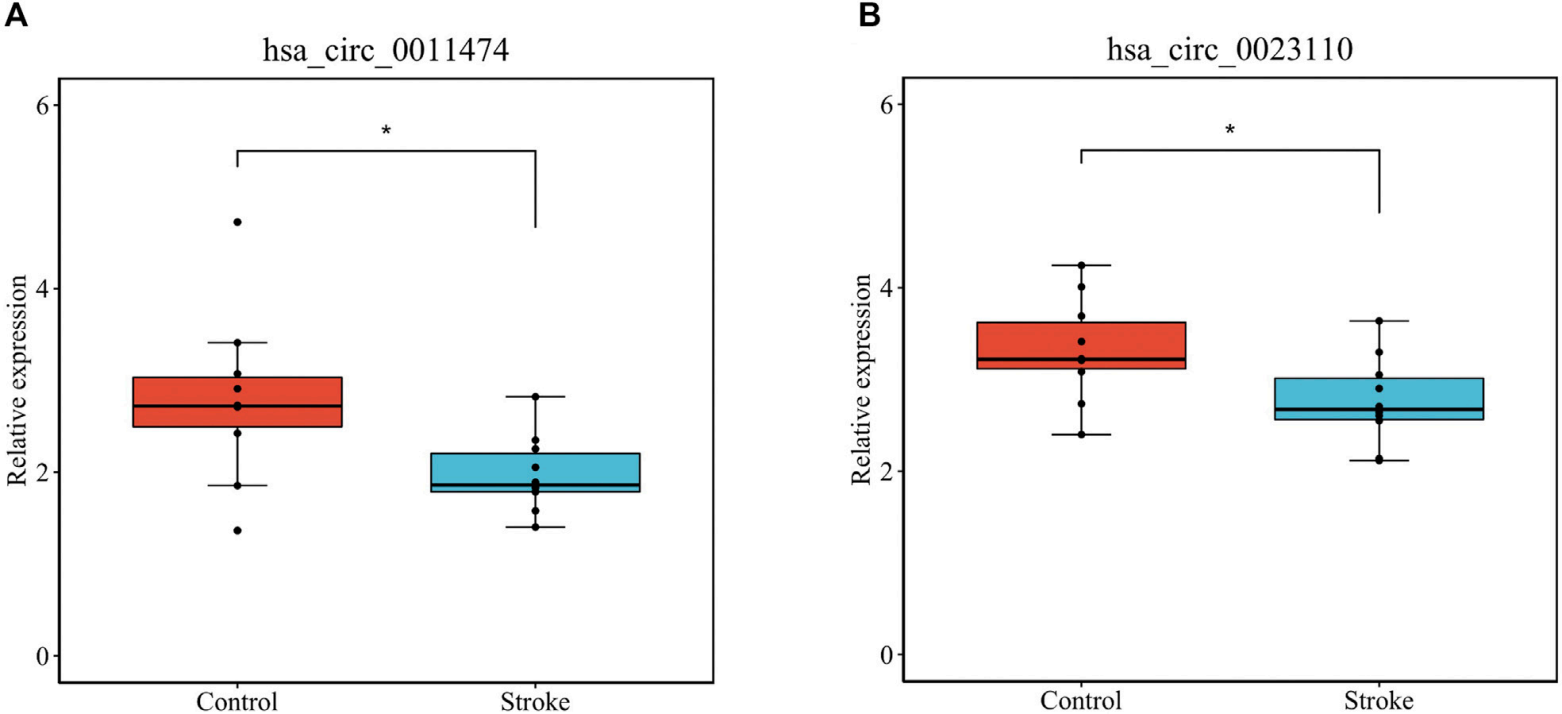
Expression levels of core circRNAs verified in GSE195442: **(A)** has_circ_0011474; **(B)** hsa_circ_0023110. (*p < 0.05)

**FIGURE 8:**
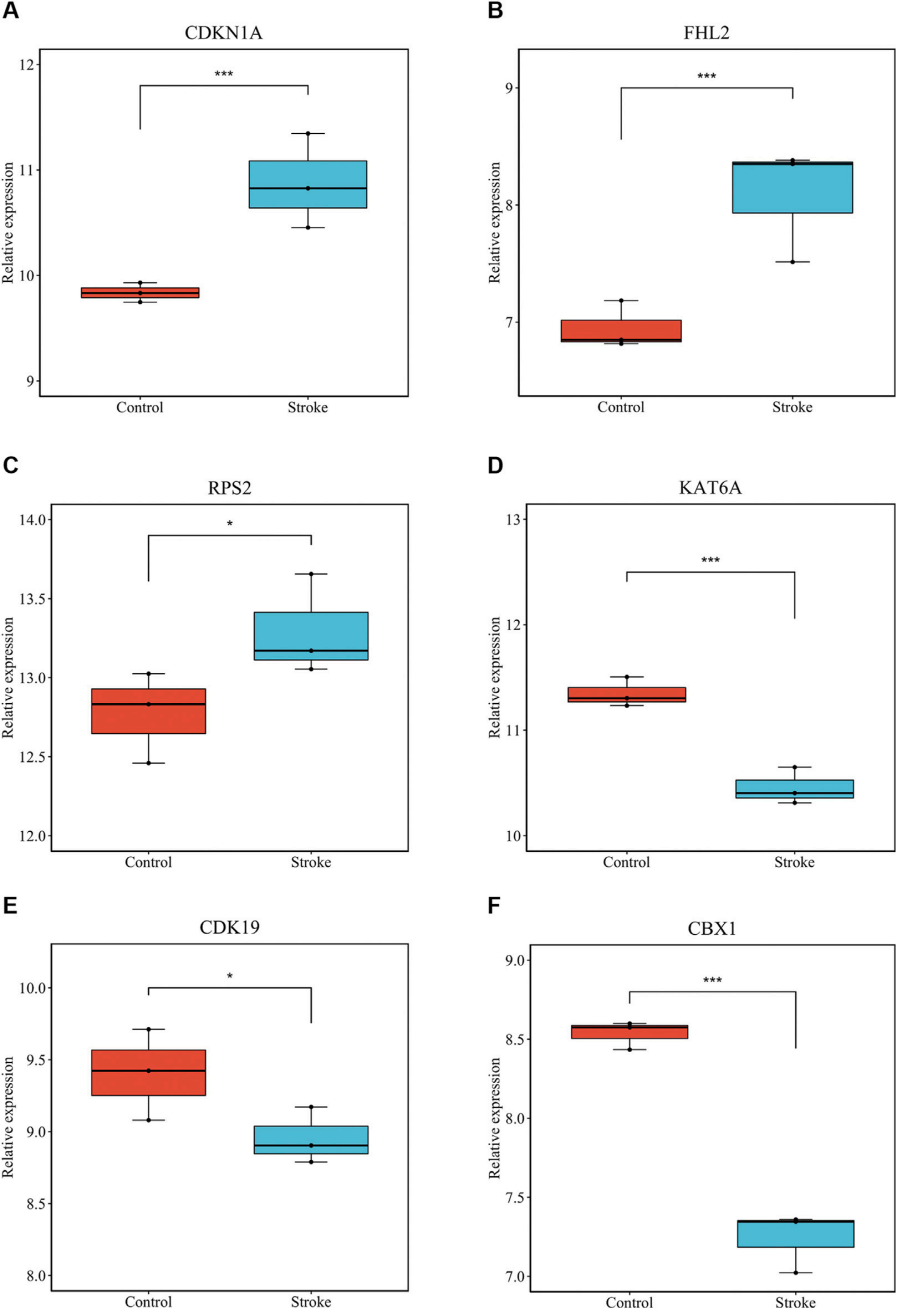
Expression levels of core mRNAs verified in GSE140275: **(A)** *CDKN1A*; **(B)** *FHL2*; **(C)** *RPS2*; **(D)** *KAT6A*; **(E)** *CDK19*; **(F)** *CBX1*. (*p < 0.05, ***p < 0.001).

**FIGURE 9:**
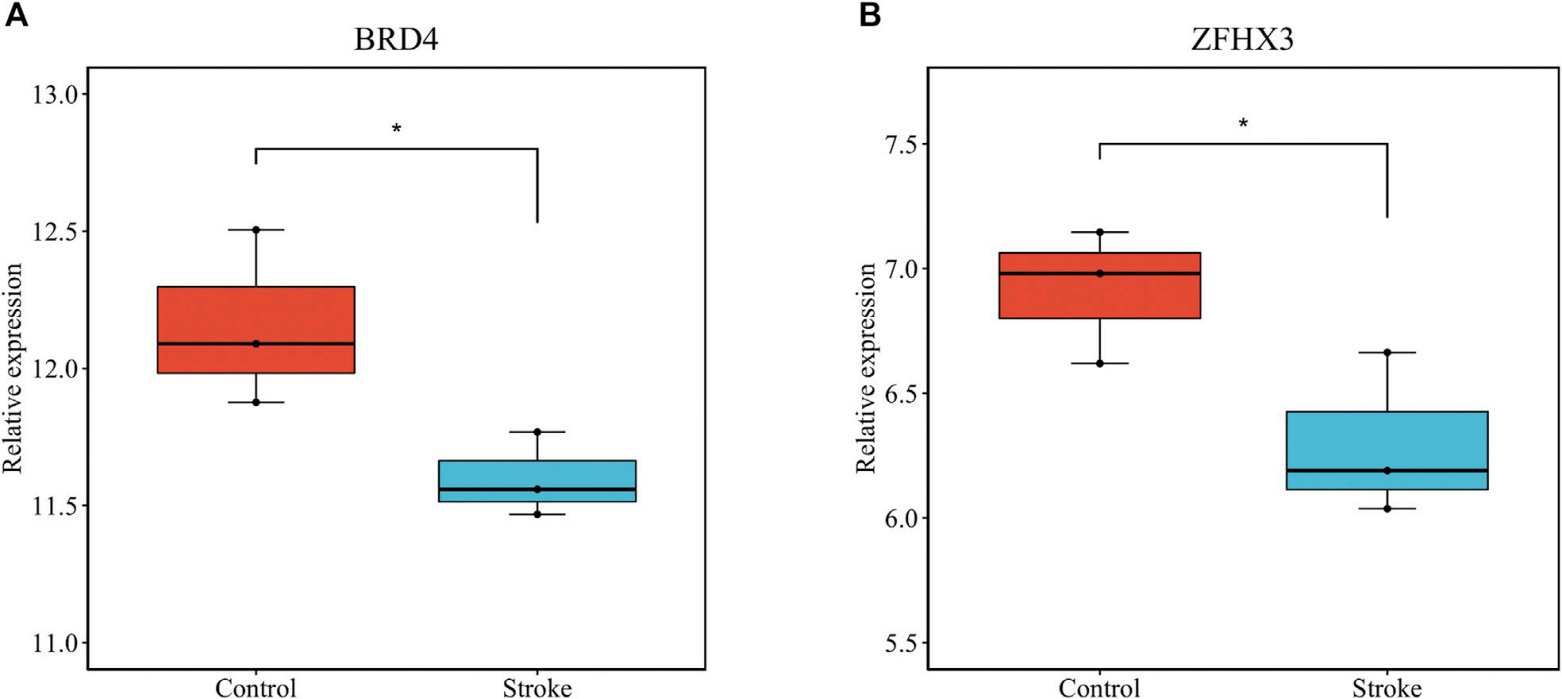
Expression levels of core mRNAs verified in GSE180470: **(A)** *BRD4*; **(B)** *ZFHX3*. (*p < 0.05).

We verified that hsa_circ_0011474 and hsa_circ_0023110, which were significantly downregulated in IS, were also significantly downregulated in the circRNA validation set GSE195442 (Figure 7). We verified that *CDKN1A*, *FHL2*, and *RPS2*, which were significantly upregulated in IS, and *KAT6A*, *CDK19*, and *CBX1*, which were significantly downregulated, had consistent expression changes in the mRNA validation set GSE140275 (Figure 8). Similarly, we verified that *BRD4* and *ZFHX3*, which were significantly downregulated in IS, were also significantly downregulated in the mRNA validation set GSE180470 (Figure 9). We did not validate any miRNAs that showed significant changes in expression consistent with GSE110993.

## 4 Discussion

Owing to the high incidence, disability, mortality, recurrence, and economic burden of stroke, the identification of specific biomarkers for the diagnosis and treatment of IS an urgent task. In recent years, an increasing number of studies have shown that circRNAs can influence the onset and progression of cardiovascular disease through multiple pathways. CircRNA-meidated ceRNA is a important form of gene regulation for circRNA, and has a great potential in disease research. However, whole transcriptome-wide screenings of circRNA-mediated ceRNA networks associated with IS are still lacking.

In this study, we used expression data downloaded from the GEO database to identify DEcircRNAs, DEmiRNAs, and DEmRNAs between IS patients and HCs. By integrating the interactions, we successfully constructed an IS-specific ceRNA network with 185 nodes and 238 edges. GO enrichment analysis showed that the DEmRNAs were mainly involved in IS-related BPs. In addition, DEmRNAs were significantly enriched in four KEGG pathways, including coronavirus disease-COVID-19. Studies have shown that patients infected with COVID-19 may be at a higher risk of IS than patients with influenza infections (Merkler et al., 2020).

To identify the core of the ceRNA network, we obtained 20 hub genes using PPI and Cytohubba analyses. Eight of these 20 genes have been reported to be associated with IS, including *ITGB3*, *CDKN1A*, *ZFHX3*, *CREBBP*, *MECP2*, *RUNX1*, *BRD4*, and *FOXO3*. Maguire et al. demonstrated that the *ITGB3* (*GPIIIa*) variant (rs5918) is associated with functional outcomes in stroke survivors (Maguire et al., 2011). Fan et al. reported that *CDKN1A/JUN* could be a robust and promising gene-pair diagnostic biomarker for IS, regulating ferroptosis during IS progression via the C9orf106/C9orf139 - miR-22-3p - CDKN1A and GAS5-miR-139-5p/miR-429-JUN axes (Fan et al., 2022). In addition, several studies have found that SNP rs7193343, located on *ZFHX3*, is associated with prognostic recovery in cardioembolic stroke, a subtype of IS (Gudbjartsson et al., 2009). Another study, Hu et al. identified SNP rs879324, located on *ZFHX3*, as an important factor influencing IS in the Chinese Han population by multifactor dimension reduction (MDR) software and least absolute shrinkage and selection operator (LASSO) logistic regression (Hu et al., 2023). Tseveleki et al. found that *CREBBP*, a gene related to the cellular stress response, was upregulated in a mouse model of permanent middle cerebral artery occlusion (Tseveleki et al., 2010). Yang et al. demonstrated through *in vivo* and *in vitro* experiments that circSCMH1 binds to the transcription factor *MECP2* and regulates the transcriptional expression of its downstream genes, thereby affecting functional recovery after stroke in mice and primates (Yang et al., 2020). Hu et al. revealed that overexpression of *MECP2* can protect mice against ischemic brain injury via disruption of the FOXO3a/SPRY2/ZEB1 signaling axis (Meng et al., 2022). Furthermore, one study showed that young rats had higher blood vessel density on day 14 post-stroke than old rats. *RUNX1* is involved in angiogenesis, and its expression level is much higher in young rats after stroke than in old rats (Buga et al., 2014). In transient IS, dBET1 ameliorates neurological dysfunction and brain injury by degrading *BRD4*, regulating inflammation and oxidative stress, and protecting the integrity of the blood-brain barrier (Liu et al., 2022). Fibrous scarring played an important role in preventing secondary expansion of tissue damage and hindering repair and regeneration after central nervous system (CNS) injury. BRD4 was involved in fibrosis in many tissues, and transforming growth factor-β1 (TGF-β1)/Smad2/3 signaling was one of the critical pathways of fibrosis. Li et al. were the first to indicate that inhibition of BRD4 delayed fibrous scarring after IS through mechanisms involving the phosphorylation of Smad2/3 (Li et al., 2022). Similarly, *FOXO3* is highly expressed in oxygen-glucose deprivation (OGD)-induced neuronal cells. Downregulation of *FOXO3* can prevent neuronal damage and inflammatory responses in OGD-induced neuronal cells by inhibiting the CITED2/IKKα axis (Deng et al., 2020). In their latest study, Deng et al. indicated that Akt/FoxO3 signaling pathway activation inhibited oxidative stress-mediated cell death through activation of autophagy. Their study supported a critical role for the Akt/FoxO3 signaling pathway in autophagy activation in stroke (Deng et al., 2023). The remaining 12 genes (*RPS2*, *CKS2*, *COMMD8*, *CDK19*, *KAT6A*, *CBL*, *CBX1*, *COPS2*, *SNX16*, *CCNG1*, *FHL2*, and *CNIH1*) have not been reported to be associated with IS.

Based on the negative regulatory miRNA-mRNA pairs corresponding to these 20 hub genes, we constructed a core sub-network of the ceRNA network. This core sub-network included 48 circRNA-miRNA pairs and 23 miRNA-mRNA pairs. For external validation, we investigated the relative expression levels of these nodes in other datasets and validated several circRNAs and genes in the core sub-network, including *CDKN1A*, *FHL2*, and *RPS2*. The downregulated nodes included hsa_circ_0011474, hsa_circ_0023110, *CDK19*, *KAT6A*, *CBX1*, *BRD4*, and *ZFHX3*. By validating the obtained circRNAs and genes, we identified a ceRNA pathway, hsa_circ_0011474 - hsa-miR-20a-5p/hsa-miR-17-5p - CDKN1A. In addition to *CDKN1A*, which has been previously reported to be associated with IS, miR-17-5p and miR-20a-5p have been identified as promising candidate biomarkers for distinguishing embolic stroke from thrombotic stroke.

*CDKN1A* (also known as P21) encodes a potent inhibitor of cyclin-dependent kinases associated with the cellular senescence pathway (Fitzgerald et al., 2015). The aging of neurons, astrocytes, and microglia in the central nervous system is involved in brain aging and the development of age-related neurological disorders (Yamazaki et al., 2016). A marker of cellular senescence, *CDKN1A* expression was significantly upregulated in the infarcted area of dissected mice 72 h after tMCAO (Torres-Querol et al., 2021). Therefore, *CDKN1A* may be involved in the occurrence and development of IS by regulating vascular cell senescence. Supporting the significance of the hsa_circ_0011474 - hsa-miR-20a-5p/hsa-miR-17-5p - CDKN1A pathway, previous studies have found that miR-20a can improve the proliferation rate of mesenchymal stem cells in the serum of IS patients by inhibiting the expression of *CDKN1A* (Kim et al., 2016). Similarly, Rastegari et al. found that *CDKN1A* was significantly associated with autism, a neurodevelopmental disorder, through the WGCNA network (Rastegari et al., 2023).

To address the lack of joint analyses of mRNA, miRNA, and circRNA in IS, this study conducted an integrated analysis of multiple GEO datasets. We combined the results of circRNA microarray expression data analysis with the results of mRNA and miRNA high-throughput sequencing data analysis and constructed ceRNA functional networks to comprehensively explore promising diagnostic biomarkers of IS and their potential mechanisms of action. Our findings improve the current understanding of ceRNA biological behaviors and their regulatory roles in IS pathogenesis. Nodes in the hsa_circ_0011474 - hsa-miR-20a-5p/hsa-miR-17-5p - CDKN1A pathway, validated in the ceRNA network, may serve as promising diagnostic biomarkers and therapeutic targets for IS.

## Data Availability

The datasets presented in this study can be found in online repositories. The names of the repository/repositories and accession number(s) can be found in the article/Supplementary Material.
