# Supplementary figures and images for: Integrated analysis of circRNA-associated ceRNA network in ischemic stroke
Source: Front Genet. 2023 May 30;14:1153518. doi: 10.3389/fgene.2023.1153518 (PMC10267749; doi:10.3389/fgene.2023.1153518)

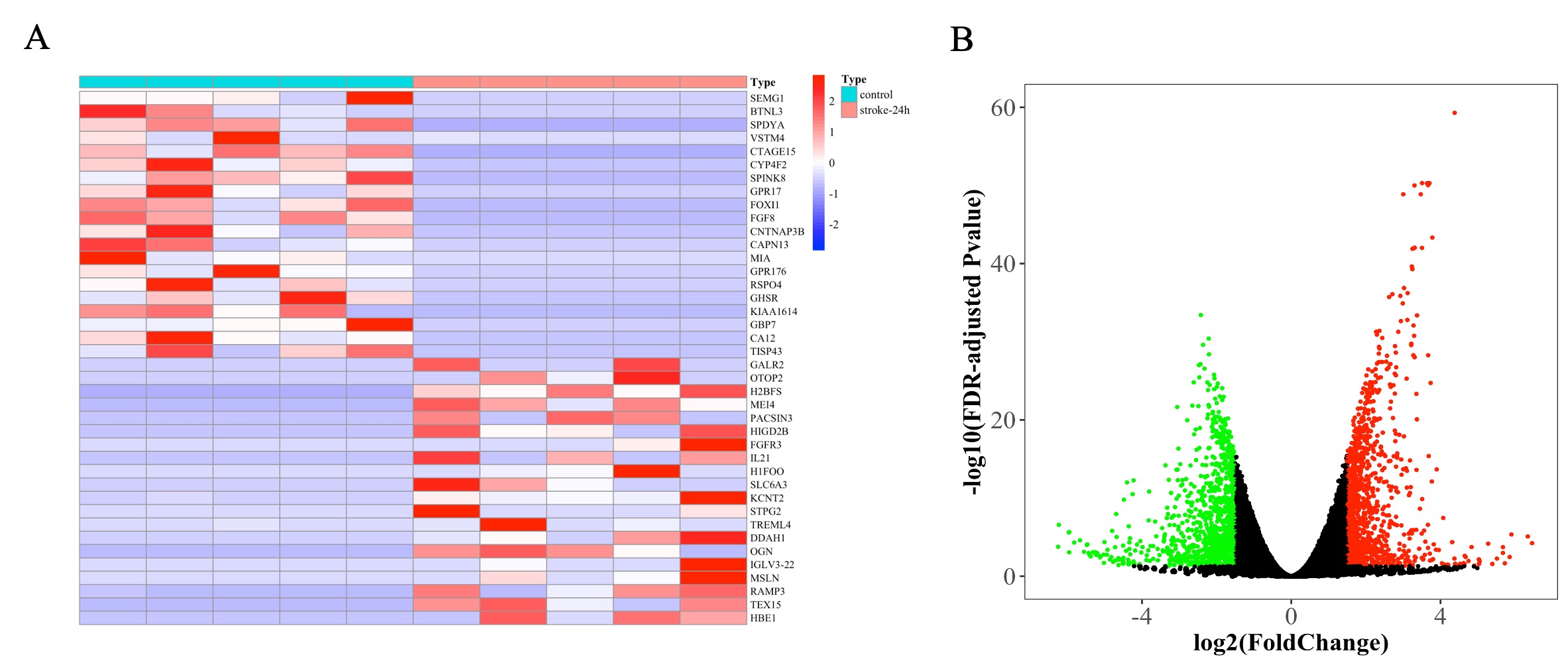

Supplement: Supplementary file 1 [file Image1.JPEG]
